# Tubulobulbar complex: Cytoskeletal remodeling to release spermatozoa

**DOI:** 10.1186/1477-7827-10-27

**Published:** 2012-04-17

**Authors:** Rahul D Upadhyay, Anita V Kumar, Malti Ganeshan, Nafisa H Balasinor

**Affiliations:** 1 Department of Neuroendocrinology, National Institute for Research in Reproductive Health, J.M.Street, Parel, Mumbai, 400012, India

**Keywords:** Tubulobulbar complex, Ectoplasmic specialization, Sperm release, Sertoli cell, Actin

## Abstract

Tubulobulbar complexes (TBCs) are actin-based structures that help establish close contact between Sertoli–Sertoli cells or Sertoli–mature germ cells (spermatids) in the seminiferous tubules of the testes. They are actin-rich push-through devices that eliminate excess spermatid cytoplasm and prepare mature spermatids for release into the tubular lumen. Just prior to spermiation, the elongated spermatid interacts with the Sertoli cell via an extensive structure comprising various adhesion molecules called the apical ectoplasmic specialization which is partially replaced by the apical TBC, on the concave surface of the spermatid head. The sperm release process involves extensive restructuring, namely the disassembly and reassembly of junctions at the Sertoli–spermatid interface in the seminiferous epithelium. Based on the presence of different classes of molecules in the TBCs or the defects observed in the absence of TBCs, the main functions attributed to TBCs are elimination of excess spermatid cytoplasm, endocytosis and recycling of junctional molecules, shaping of the spermatid acrosome, and forming transient anchoring devices for mature spermatids before they are released. This review summarizes the recent findings that focus on the role of TBCs in cell cytoskeleton restructuring during sperm release in the testes and the molecular mechanism involved.

## Background

Spermatogenesis is a complex process of differentiation leading to the generation of haploid, highly specialized spermatozoa from diploid stem cells [1]. It is associated with extensive restructuring of junctions at Sertoli–Sertoli cell, Sertoli–germ cell and Sertoli–basement membrane interfaces [2].

One of the two testis-specific actin-based structures is the ectoplasmic specialization (ES), an actin-based atypical adherens junction between adjacent Sertoli cells at the blood–testis barrier (BTB) termed as basal ES) and between Sertoli cells and spermatids near the luminal surface of the tubule termed as apical ES [3]. The ES is composed of hexagonal bundles of actin filaments sandwiched between the endoplasmic reticulum and Sertoli cell plasma membrane. In the rat testis, the apical ES first appears between Sertoli cells and round spermatids in late stage VIII and early stage IX. The basal ES is part of the junctional complex forming the BTB between adjacent Sertoli cells throughout the seminiferous epithelium cycle. The primary function of the ES is to facilitate germ cell movement, with an additional anchoring function via adhesion molecules such as integrin–laminin and nectinafadin complexes that helps retain germ cells (mainly spermatids) in the seminiferous epithelium until spermiation [3].

Another testis-specific structure is the tubulobulbar complex (TBC), which replaces the apical ES. Similar to the rat, TBCs in other mammals appear at a particular developmental stage of the seminiferous epithelium cycle [4]. Because of the difficulties in observing and identifying basal TBCs, very little information is available about their structure and importance, whereas apical TBC formation, being an inevitable event during sperm release, has been studied to a larger extent. In this review, we present a detailed discussion of apical TBCs during spermatogenesis.

### Structural details of TBCs

TBCs are cytoplasmic evaginations of mature elongated spermatids. They penetrate into the surrounding Sertoli cell cytoplasm and are composed of both tubular and bulbous portions. The tube is surrounded by fine filaments of actin and the bulb is flanked by smooth endoplasmic reticulum. Numerous double membrane vesicles are present in the vicinity of these TBCs. TBCs are very interesting specialized structures located between Sertoli cells and spermatids, appearing in the last few days prior to sperm release. They form in large numbers at the beginning of stage VII of the rat seminiferous epithelium cycle [5] as the spermatids move towards the lumen of the seminiferous epithelium from the deep recesses of the Sertoli cell. TBCs have been studied in the testes of ten mammalian species, namely opossum, vole, guinea pig, mouse, hamster, rabbit, dog, ram, monkey, and human and up to 24 TBCs are formed per spermatid [4,6]. However, TBCs have been most extensively studied and characterized in rats. In rats, the TBCs that are located near the BTB are termed basal TBCs, while those at the ventral (or concave) side of the spermatid heads of step 18 and 19 spermatids attached to the Sertoli cell are termed as apical TBCs [7].

Russell [5] analyzed the ultrastructure of TBCs and performed a time-course study of the appearance and degradation of TBCs. In rats, the length of the entire complex is 3–5 μm. In stage VI, the spermatid heads are embedded within the deep recesses of Sertoli cells, and in early stage VII, the spermatids move towards the lumen and the TBCs start to form. The complexes at this stage are short tubular projections (0.1–0.2 μm in length) of the spermatid projecting towards the Sertoli cells. The bulbous region develops as a dilated component in the mid-region of the tube, facing the smooth endoplasmic reticulum of the Sertoli cell. By mid-stage VII, large vacuoles containing double membrane-bound vesicles accumulate around the clustered bulbs, which fuse with the lysosomes and are degraded or selectively recycled to the plasma membrane [5,8]. In late stage VII and early stage VIII, the ESs get displaced and the tubular portions of old TBCs start getting resorbed, while new TBCs start forming as evidenced by bristle-coated pits in Sertoli cell invaginations and short opposing evaginations of the spermatid. This suggests that several generations of TBCs are formed and resorbed prior to sperm release [5,8].

### Resemblance of TBCs to podosomes

TBCs resemble podosomes present in other systems. Podosomes are dot-like contacts that a cell makes with the extracellular matrix. They are known to occur specifically in monocyte-derived haematopoetic cells including macrophages, osteoclasts, and dendritic cells [9], while TBCs occur only in the seminiferous tubules of the testis. Podosomes comprise a core of F-actin and actin-associated proteins embedded in a ring structure of integrins and integrin-associated proteins. Actin-associated proteins such as cortactin, talin, and vinculin link integrins to the actin cytoskeleton [9]. Similarly, TBC formation is shown to be generated by the assembly of the actin network involving the action of N-WASP (Neural Wiskott-Aldrich syndrome protein), the Arp2/3 (actin-related protein) complex, cortactin, and dynamin [10]. Podosomes are dynamic in nature: they are repeatedly constructed and destroyed and hence are thought to be suitable for transient adhesions during cell motility. In addition to podosomes, a similar actin-based structure exists in yeast during budding events, highlighting the involvement of the actin cytoskeleton in clathrin-mediated endocytosis, which is likely to be conserved across eukaryotes [11]. Similar observations seen in TBCs are supported by the fact that when adherent ESs between the Sertoli cell and spermatid start to disappear at stage VII, TBCs concomitantly appear in the areas where the ESs disappear [12].

### Cytoskeleton associated with TBC

#### Actin

Like podosomes, TBCs comprise a core of F-actin around the tubular invagination. F-actin (filamentous actin) is a polymer and the basic unit of microfilaments. It is an assembly of G-actin (globular actin) subunits and can produce rapid changes in cytoskeletal dynamics through polymerization, depolymerization, severing, capping, bundling, and branching with the help of actin-associated proteins. Generally, F-actin is found abundantly at sites of cell–cell contact and hence localized to the BTB (basal ES and TBCs) and in the adluminal compartment (apical ES and TBCs). However, the arrangement of actin in the ES and TBC differs. It is packed in hexagonal arrays in the ES, while it appears as a branched network surrounding the tubular portion of TBCs [13]. Use of cytochalasin D, an inhibitor of actin polymerization, showed that TBCs did not develop normally since they lacked both tubular and bulbous components and the affected spermatozoa contained an abnormally large amount of cytoplasm. This suggested that actin is important in the formation of the tubular portion of the TBC and retention of excess cytoplasm was due to filament disruption [14].

#### Microtubules

Microtubules are polymers of α and β tubulin mainly involved in the transport of vesicles and other cargo by motor proteins that move along them. Immunofluorescence studies showed a close relationship between the microtubules and the heads of elongate spermatids [13]. Also, dynein [15] and kinesin [16], the two motor proteins commonly involved with transport along microtubules are associated with the ES bound to elongated spermatids.

Thus, spermatid translocation during spermiogenesis is hypothesized to occur via microtubule-based transport of the apical ES. The role of microtubules has also been suggested in apical ES restructuring, which is later replaced by apical TBCs [13]. This speculation is based on studies on focal adhesion disassembly by Ezratty *et al.*[17] who showed that the molecular components are similar to that of apical ES [13,17]. Microtubules have been implicated in focal adhesion disassembly through clathrin, dynamin, FAK, and Erk signaling-mediated endocytosis in TBCs [17,18].

#### Intermediate filaments

Keratin and vimentin are important intermediate filaments present in the rat testis in neonates and adults, respectively. In adult rat testis, vimentin shows stage-specific localization in pre- and post-spermiation stages as long and short apical projections, respectively. However, during spermiation (stages VII–VIII in rat), vimentin has only perinuclear localization. There was no localization of vimentin in TBCs. However, studies from our lab have shown that an important cytolinker of intermediate filaments called plectin localizes to TBCs [19]. Plectin, in the TBCs, could be involved in the progression of tubule formation as demonstrated in A7r5 rat smooth muscle, whereas plectin was demonstrated to be involved in the formation of podosomes [20].

### Proteins associated with TBC formation

Various proteins involved in actin bundling, polymerization, and stability are found in podosomes, which resemble TBCs. Hence, there have been studies to see if these proteins are present on TBCs and if they play a role in their formation. The Arp2/3 complex is a key regulator of actin polymerization and branching that promotes filament assembly through enhanced nucleation of actin subunits [21]. Arp3 (actin-related protein 3) is localized to the concave side of the spermatid head, the area in which apical TBCs are located [22]. Actin-binding proteins such as espin, for actin bundling, and vinculin, which shows actin crosslinking activity, are present on TBCs as shown by immunofluorescence staining [23]. High paxillin expression was detected in TBCs with paxillin and vinculin colocalization seen in close association with the actin network suggesting interactions between the two proteins. Paxillin is generally involved in turnover of actin networks [21]. Cofilin-1 (also known as non-muscle cofilin), which is involved in actin depolymerization, is found in apical TBCs and is thought to increase actin turnover in TBCs [12]. Young *et al.*[24] have shown the presence of N-WASP (Wiskott–Aldrich syndrome protein) and cortactin, key components present in podosomes, are also present on TBCs. N-WASP is a key regulator of the Arp2/3 complex, which in turn generates new actin filament branches from pre-existing filaments, thereby forming a three-dimensional actin network in which filaments elongate from their barbed ends positioned at the plasma membrane [24]. Blockage of N-WASP by Wiskostatin, a chemical inhibitor, results in mis-orientation of step 19 spermatids [22]. CR16, a member of WASP-interacting proteins, is highly expressed in the testis, especially in Sertoli cells. In CR16 male knockout mice, sperm head morphology was affected along with diminished fertility [25]. All these data suggest that actin and its regulatory proteins in TBCs are also involved in sperm head morphology.

Recent studies have shown actin and actin-regulatory proteins to be involved in endocytosis [26]. Since TBCs are also endocytic devices, various endocytic proteins are known to be localized at the site of their formation [10]. Amphiphysin 1 is concentrated at the luminal surface of seminiferous tubules at stage VIII during rat spermatogenesis. It is involved in clathrin-mediated endocytosis and also in regulation of actin cytoskeleton [27]. Amphiphysin 1 is important for actin polymerization during phagocytosis as demonstrated by colocalization studies with actin, vinculin, and cortactin around TBCs [28]. Dynamin family proteins are known to be involved in the process of endocytosis. Dynamin 2 and 3 are implicated in vesicle formation and clathrin-mediated endocytosis [29] and are shown to be involved in tubular morphogenesis in TBCs [30,31]. Clathrin-lattice machinery is known to be triggered by assembly proteins (APs), namely AP1 and AP2 [32,33]. Similarly, the non-neuronal homolog of AP180 protein, PICALM, plays a significant role in clathrin internalization machinery [34]. However, there are no reports describing their localization and function in TBCs. In addition, over 50 proteins are associated with clathrin-mediated endocytosis [35]. Some of these are likely to be involved in TBC formation and function [10,28,30-32]

Recently, the actin regulator Eps8 (epidermal growth factor receptor pathway substrate 8), which controls actin-based motility by capping barbed ends of actin filaments [36] and has been detected in both Sertoli and germ cells and localized in apical TBCs, has been reported to play an important role in maintaining germ cell adhesion and BTB integrity in rat testes [36]. Eps 15 homology domain containing protein 1 (EHD1) regulates endocytic trafficking and recycling of membrane components during spermatogenesis. Knockout models of EHD1 male mice have shown acrosomal defects, mis-orientation of spermatids and phagocytosis of failed elongated spermatids [37]. Detailed description and function of proteins found to be localized in TBCs are presented in Table 1.

**Table 1:** Proteins localized at the sperm release boundary

**PROTEIN NAME**	**FUNCTON**	**REFERENCE**
Arp3	Formation of branched actin network in TBCs	D’Souza *et al.*[38]
**Actin-binding proteins**		
Espin	Actin bundling	Guttmann *et al.*[24] Young *et al.*[10]
Vinculin	Actin cross-linking	
Cortactin	Actin depolymerization: increases actin turnover in TBCs	Young *et al.*[10]
Cofilin	Arp2/3 complex activation	Guttmann *et al.*[12]
Eps8	Actin capping; regulation of bundling and Rac-GTPase: formation of actin bundles and network	Lie *et al.*[31]
Paxillin	Turnover of actin network	Mulholland *et al.*[23]
N-WASP	Arp2/3 complex activation	Young *et al.*[10]
Profilin IV	Assembly–disassembly of F-actin, presumably present at tubulobulbar extensions of Sertoli cells	Obermann *et al.*[39]
**Adhesion molecules**		
α6 β1-integrin	Complex associated with disengagement during spermiation	Beardsley *et al.*[40]
Nectin 2	Expressed only in Sertoli cells and stabilizes association of TBC–Sertoli cell adherens complex	Kierzenbaun *et al.*[41], Mueller *et al.*[42]
Nectin 3	Associated with actin and espin, forms a hetero-trans dimer with nectin 2	Lee *et al.*[3], Inagaki *et al.*[43]
Afadin	Nectin–actin linker protein	Guttmann *et al.*[24]
**Endocytosis**		
Clathrin	Forms a layer around endocytic vesicles forming clathrin-coated pits	Young *et al.*[10]
Amphiphysin 1	Co-operates with dynamin 1 in clathrin-mediated endocytosis; also involved in actin dynamics	Kusumi *et al.*[28]
Dynamin 2	Pinching off vesicles from the parent membrane and formation of tubular protrusions	Kusumi *et al.*[28]
Dynamin 3		Vaid *et al.*[16]
Early endosomal antigen (EEA)	Marker for early endosomes, linked to internalization–degradation pathway of TBCs	Young *et al.*[24]

Adhesion molecules such as nectin 2, nectin 3, and α6 integrin have been reported in apical TBCs and appear to be concentrated at their ends [24]. Guttman *et al.*[37] have shown that TBCs develop in regions previously occupied by ESs by studying the localization of molecular markers for ES, namely espin, myosin VII, and Keap I, all of which also localize to the TBCs. Afadin, which binds nectin to actin filaments, is found in ESs and TBCs; when a step-wise progression of afadin staining was investigated during maturation of step 18–19 spermatids, staining of ESs along the dorsal curvature of spermatids gradually appeared to decrease in intensity, whereas an increase in staining intensity was observed around TBCs where it formed a fingerlike staining pattern [44].

### Estrogen regulation in TBCs

Current knowledge of hormonal regulation during spermatogenesis supports the proposition that both testosterone and FSH have similar and independent actions that are essential for quantitatively normal spermatogenesis in adulthood [45]. Testosterone is also known to maintain the binding competency of Sertoli cells and Sertoli cell–spermatid junctional interaction [46]. The process of spermiation, involving formation of TBCs and subsequent release of sperm into the tubular lumen, is known to be responsive to hormones. The major hormonal regulators of spermiation are FSH and androgens [38]. It has been shown that in rats, suppression of either FSH, androgen, or both causes spermiation failure. The combined suppression of FSH and testosterone had a significantly greater effect on spermiation than the two alone. This failure appears to be only due to the inability of the spermatid to disengage from the Sertoli cell cytoplasm, the formation of ES and TBCs seems unaffected. In this model, an adhesion molecule, β1-integrin, is likely to mediate spermatid disengagement as indicated by its localization around retained spermatids by Beardsley *et al.*[40,47]. Also, testosterone capsules implanted in hypophysectomized rats, which led to decrease in intratesticular testosterone concentration, prevented TBCs from forming in many late spermatids during their release. These spermatids displayed an accumulation of cytoplasm (swelling) in the perinuclear region of the head in turn suggesting a role of testosterone or its metabolites in TBC formation [48]. Our studies using a model that involved administration of exogenous estradiol to rats leading to suppression of FSH and intratesticular testosterone, and a concomitant increase in intratesticular estrogen, showed marked spermiation failure. The failure was likely due to the absence of TBCs, shown in electron micrographs, and lack of localization of F-actin (which is a marker for TBCs) [49,50]. Looking at the molecular aspects, Arp2/3 complex transcript levels were unaltered in the FSH and T suppression model, whereas, transcripts of one of the subunits of Arp2/3 complex, namely Arpc1b, involved in complex stabilization, was found to be reduced in the exogenous estradiol model [51,52]. These observations could indicate that formation of TBCs is most likely mediated by estrogen rather than by FSH and androgens; however, the molecular mechanism of estrogen action in TBC formation is yet to be investigated.

### Functions of TBCs

#### Attachment device between Sertoli cells and spermatids

In the rat, TBCs are first observed at early stage VII of the seminiferous epithelium cycle as short tubular invaginations. As the spermatids progress towards sperm release, the TBCs become slightly larger with a bulbous structure; this structure is observed to become smaller and more elongated in mid-stage VII. TBCs continue to be formed and resorbed through early stage VII up to sperm release at late stage VIII [4]. Additionally, abnormally shaped sperm that are not released are seen to have intact ES and TBCs associated with them. [5]. Loss of ES and its signal abolition may expose spermatids to the tubular lumen: TBCs may then serve to anchor spermatids to the Sertoli cell apical cytoplasm. Formation of a number of TBCs at the spermatid head indicate that TBCs are capable of holding or anchoring the head in proper place even though there appears to be less contact between the Sertoli cell and spermatid [4]. Electron microscopy studies by Russell and Clermont, [48] suggested that TBCs are anchoring devices, anchoring the head of spermatids to the apical cytoplasmic processes of the Sertoli cell, while their dissolution contributes, in part, to spermiation. However, our studies, as well as studies with amphiphysin knockouts [28] demonstrated the absence of TBCs and an increase in the number of retained spermatids, which suggested that TBCs are not involved in adhesion/attachment. However, it can be argued that TBCs could be transient attachment devices, as discussed by O’Donnell *et al.*[7], whereby during spermiation, step 18–19 spermatids are extended out into the tubular lumen and are subjected to shear forces from the seminiferous tubule fluid flow. The transient attachment of spermatids to Sertoli cells through TBCs may be sufficiently strong to counter this force.

#### Endocytic device of Sertoli cells for elimination of spermatid cytoplasm and shaping of spermatid acrosome

Several studies suggest a process of active formation and resorption of TBCs until sperm release into the tubular lumen. The resorption of one large 4 μm complex implies the loss of 12 μm^2^ of spermatid membrane and 4 μm^3^ of spermatid cytoplasm [5]. This implies a substantial portion of spermatid membrane and cytoplasm is absorbed by the Sertoli cell from the maturing spermatid and phagocytosed. There is substantial evidence of phagocytic activity in the Sertoli cells in and around the terminal ends of TBCs. The terminal ends are associated with vesicles, some of which are associated with lysosomes, based on acid phosphatase activity. Phagocytic vacuoles observed in Sertoli cells near the TBCs and the tubular portions of the TBCs also show acid phophatase activity [5]. In addition, lysosomal markers LAMP-1 and SGP1 are also localized to vesicles associated with the terminal ends of TBCs [44]. This may be a significant function of TBCs.

TBCs may also play a role in the remodeling of the acrosome prior to sperm release. Immunoreactivity towards MN7, an acrosomal glycoprotein, is seen in TBCs. A proportion of TBCs protruding from the acrosomes of step 19 spermatids showed PAS staining, conventionally used to visualize the acrosome. Based on these observations, it is hypothesized that some acrosomal contents protrude into some TBCs during steps 18 and 19, and may be phagocytosed by Sertoli cells, thereby eliminating excess acrosomal content leading to the shaping of the spermatid acrosome [53].

#### Intercellular ES and adhesion junction disassembly and internalization

In addition to the degradation of the contents of the endocytic vesicles by lysosomes, some of these contents, namely junctional molecules, are internalized and recycled back to the plasma membrane of the Sertoli cell [24]. Guttman *et al.*[44] and Young *et al.*[8,24] provide evidence that TBCs are involved in junction recycling. TBCs develop in regions previously occupied by ES and contain molecular markers for ES. Integrins, membrane adherens proteins nectin 2, and nectin 3 appear in the vesicles near TBCs; further, nectin 3 is observed in spermatids attached to Sertoli cells but not in the released spermatids [44]. Also, the presence of double-membrane vesicles at the ends of TBCs supports the hypothesis that adhesion domains of Sertoli cells and spermatids may be internalized together in TBCs [44].

As stated earlier, absence of TBCs following estradiol treatment presumably resulted in failed spermiation [49,50]. It can be hypothesized that an inability to form TBCs would result in an inability to remove Sertoli cell membranes and associated cell adhesion molecules from around mature spermatids, thereby causing spermiation failure: this is supported by the absence of endocytic vesicles and retention of α6β1 integrin and vinculin around failed step 19 spermatids [40]. Thus the removal of adhesion molecules by means of TBCs may be important for normal sperm release.

**Figure 1:**
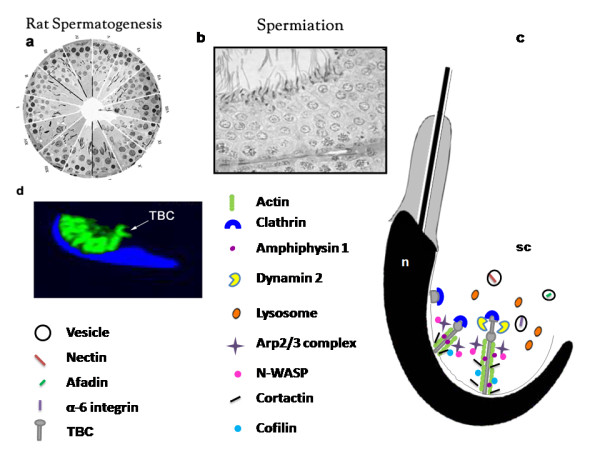
**Schematic drawing of TBC assembly during spermiation**. (a) A composite image of 14 stages of rat spermatogenesis. (b) Enlarged view of stage VIII showing mature (step 19) spermatids at the luminal edge of the seminiferous tubules about to be released. (c) Dynamics of TBC formation and function depicting endocytic proteins, actin-regulating proteins and adhesion molecules (sc, Sertoli cell cytoplasm; n, spermatid nucleus). (d) A magnified fluorescent image of a single spermatid head (nucleus stained blue with DAPI) along with F-actin (stained green) localization along the TBCs.

A diagrammatic representation of the TBC formation machinery is depicted in Figure 1.

## Conclusions

During the last few years, knowledge about TBCs has progressed substantially. However, there are still many questions left unanswered, such as what signals ES dissolution and/or removal and TBC formation, is ES dissolution the signal for TBC formation or vice-versa, is there crosstalk between ES and TBC and several others which are very basic. The genesis of TBCs is still not very clear and the complex signals that specify the exact time of formation and meticulous dissolution of TBC need to be explored. Moreover, the hormonal regulation of TBC formation is not very well understood and needs to be investigated.

Studies on the testosterone and FSH suppression model demonstrated no effect on TBC formation, while our studies using exogenous estradiol treatment leading to a decrease in testosterone and FSH with a concomitant increase in intratesticular estrogen demonstrated the absence of TBCs [16,30]. Hence, our studies suggest that TBC formation is negatively regulated by estrogen and T/FSH has no role in the genesis of TBC. However, it may be possible that in the testosterone/FSH suppression model, the decreased concentration of T and FSH in the testis may be sufficient to maintain TBC formation. The insight into TBC structure and function will help in delineating the sophisticated process of spermiation, thus answering some of the unsolved questions in the development of a contraceptive regime. The signaling cascade of TBC formation, involving actin regulating pathways, endocytic pathways and their interconnections is not yet established.

## Competing interests

The authors declare that they have no competing interests.

## Authors’ contributions

RDU made the initial observation, designed, composed figures and executed majority of the writing of the manuscript. NHB participated in designing, correcting and analyzing the manuscript in association with RDU. AVK and MG helped in composing the figures and writing a part of the manuscript. All authors read and approved the final manuscript.
